# Supplementary material for: Effects of Hydrothermal Processing Duration on the Texture, Starch and Protein In Vitro Digestibility of Cowpeas, Chickpeas and Kidney Beans
Source: Foods. 2021 Jun 18;10(6):1415. doi: 10.3390/foods10061415 (PMC8234845; doi:10.3390/foods10061415)
Supplement: Supplementary file 1 [file foods-10-01415-s001.zip › foods-1227346-supplementary.pdf]

*Supplementary Materials*

# Effects of hydrothermal processing duration on the texture, starch and protein *in vitro* digestibility of cowpeas, chickpeas, and kidney beans

**Prit Khrisanapant [1,2], Sze Ying Leong [1,2], Biniam Kebede [1], and Indrawati Oey [1,2] ***

[1] Department of Food Science, University of Otago, PO Box 56, Dunedin 9054, New Zealand; prit.khrisanapant@outlook.com (P.K.); biniam.kebede@otago.ac.nz (B.K.); sze.leong@otago.ac.nz (S.Y.L.)

[2] Riddet Institute, Private Bag 11 222, Palmerston North 4442, New Zealand

* Correspondence: indrawati.oey@otago.ac.nz; Tel: +64-3-479-8735

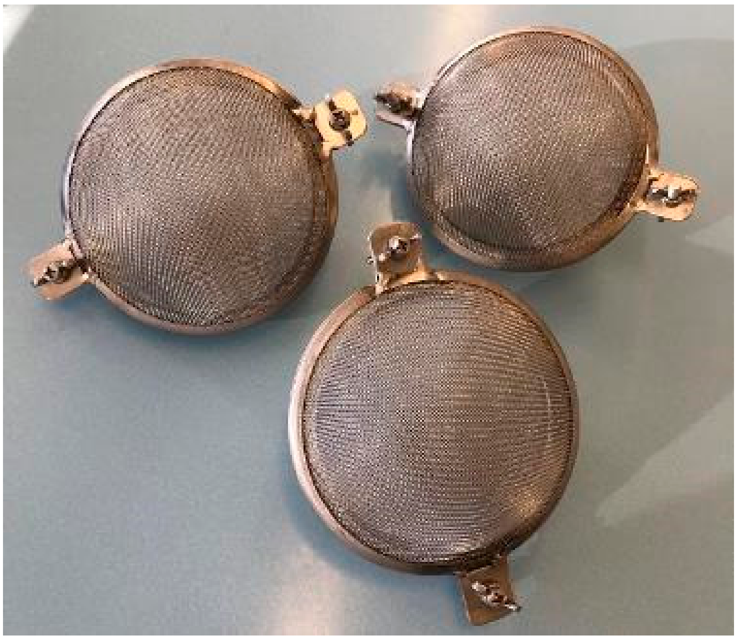

**Figure S1.** Picture of stainless-steel sieves used during hydrothermal processing of legumes.

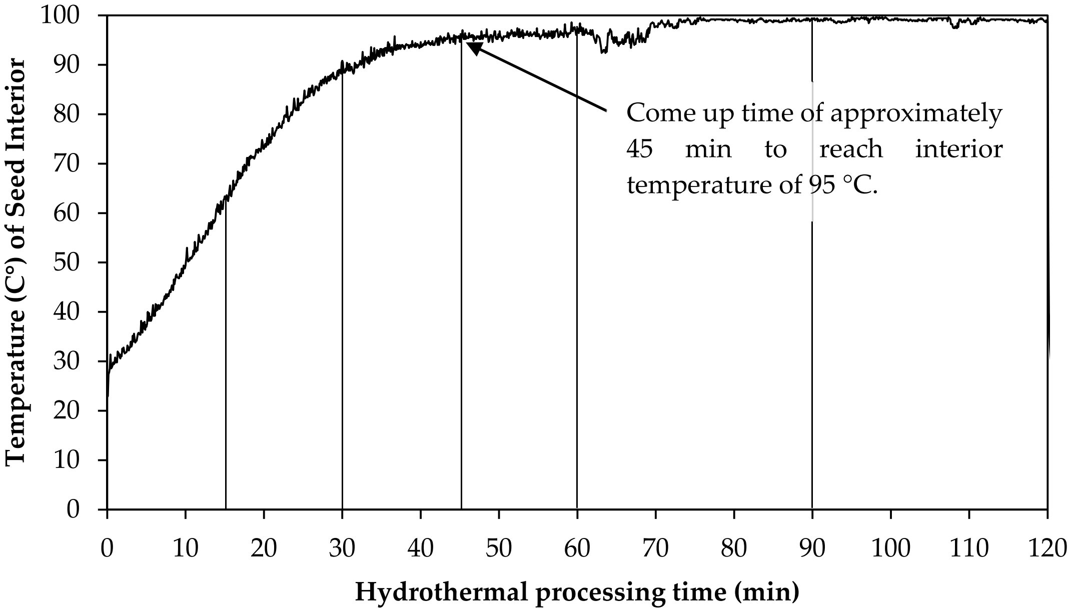


**Figure S2**. Time-temperature profile of seed interior during hydrothermal processing.

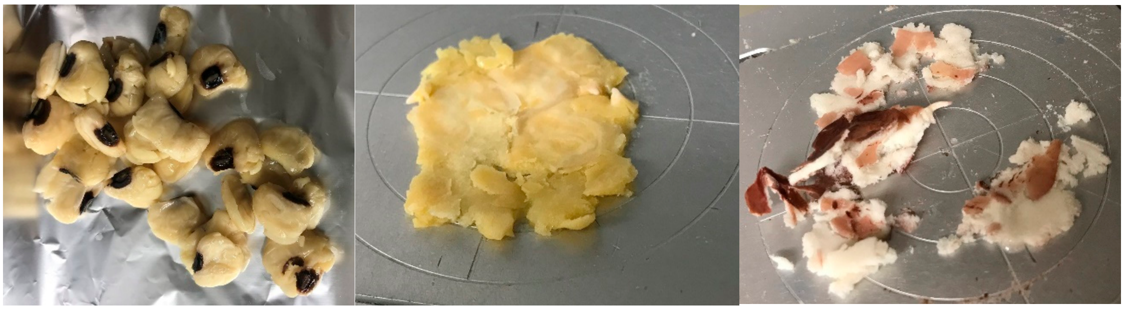

**Figure S3.** From left to right: soaked cowpeas, boiled chickpea and boiled kidney bean after compression to 90% of its strain using a texture analyser (TA.HDplus texture analyser platform using a 50 mm diameter cylinder probe (P/50)).

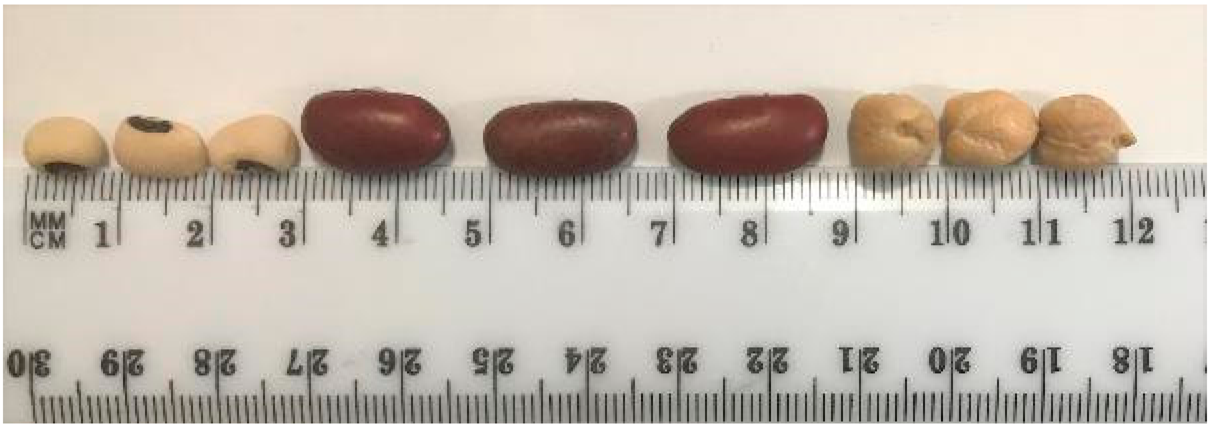


**Figure S4.** Picture of cowpea (left), kidney bean (middle), and chickpea (right) with different sizes used in this study.

**Table S1.** Starch, protein, lipid and moisture content of chickpeas, cowpeas and kidney beans.

| Legume | Starch (% DW) | Protein (% DW) | Lipid (% DW) | Moisture (% FW) |
|---|---|---|---|---|
| Cowpeas | 49.49 | 21.58 | 3.46 | 9.13 |
| Chickpeas | 47.25 | 16.74 | 7.73 | 8.27 |
| Kidney beans | 51.07 | 16.39 | 3.59 | 12.52 |

% DW represents % value in dry weight. % FW represents % value in fresh weight.
